# Modifiable Risk Factors for Common Ragweed (*Ambrosia artemisiifolia*) Allergy and Disease in Children: A Case-Control Study

**DOI:** 10.3390/ijerph15071339

**Published:** 2018-06-26

**Authors:** Maureen Agnew, Ivana Banic, Iain R. Lake, Clare Goodess, Carlota M. Grossi, Natalia R. Jones, Davor Plavec, Michelle Epstein, Mirjana Turkalj

**Affiliations:** 1 School of Environmental Sciences, University of East Anglia, Norwich NR4 7TJ, UK; m.agnew@uea.ac.uk (M.A.); c.goodess@uea.ac.uk (C.G.); C.Grossi-sampedro@uea.ac.uk (C.M.G.); n.jones@uea.ac.uk (N.R.J.); 2 Department Children’s Hospital Srebrnjak, Srebrnjak 100, 10000 Zagreb, Croatia; cosic@bolnica-srebrnjak.hr (I.B.); plavec@bolnica-srebrnjak.hr (D.P.); turkalj@bolnica-srebrnjak.hr (M.T.); 3 Division of Immunology, Allergy and Infectious Diseases, Department of Dermatology, Währinger Gürtel 18–20, Room 4P9.02, 1090 Vienna, Austria; michelle.epstein@meduniwien.ac.at

**Keywords:** ragweed, allergy, climate change, rhinoconjunctivitis, sensitization, *Ambrosia artemisiifolia*

## Abstract

Ragweed allergy is a major public health concern. Within Europe, ragweed is an introduced species and research has indicated that the amounts of ragweed pollen are likely to increase over Europe due to climate change, with corresponding increases in ragweed allergy. To address this threat, improving our understanding of predisposing factors for allergic sensitisation to ragweed and disease is necessary, specifically focusing upon factors that are potentially modifiable (i.e., environmental). In this study, a total of 4013 children aged 2–13 years were recruited across Croatia to undergo skin prick tests to determine sensitisation to ragweed and other aeroallergens. A parental questionnaire collected home environment, lifestyle, family and personal medical history, and socioeconomic information. Environmental variables were obtained using Geographical Information Systems and data from nearby pollen, weather, and air pollution stations. Logistic regression was performed (clustered on school) focusing on risk factors for allergic sensitisation and disease. Ragweed sensitisation was strongly associated with ragweed pollen at levels over 5000 grains m^–3^ year^−1^ and, above these levels, the risk of sensitisation was 12–16 times greater than in low pollen areas with about 400 grains m^–3^ year^−1^. Genetic factors were strongly associated with sensitisation but nearly all potentially modifiable factors were insignificant. This included measures of local land use and proximity to potential sources of ragweed pollen. Rural residence was protective (odds ratio (OR) 0.73, 95% confidence interval (CI) 0.55–0.98), but the factors underlying this association were unclear. Being sensitised to ragweed doubled (OR 2.17, 95% CI 1.59–2.96) the risk of rhinoconjunctivitis. No other potentially modifiable risk factors were associated with rhinoconjunctivitis. Ragweed sensitisation was strongly associated with ragweed pollen, and sensitisation was significantly associated with rhinoconjunctivitis. Apart from ragweed pollen levels, few other potentially modifiable factors were significantly associated with ragweed sensitisation. Hence, strategies to lower the risk of sensitisation should focus upon ragweed control.

## 1. Introduction

Pollen associated with common ragweed (*Ambrosia artemisiifolia*; hereafter referred to as ragweed), an herbaceous plant native to North America, is allergenic. Ragweed represents a particular threat to allergy sufferers because each plant can produce up to one billion pollen grains annually and the pollen is a potent allergen [1]. In the U.S., over 26% of the population is sensitised to ragweed [2]. Ragweed has been introduced into Europe, which has caused an invasion event [3]. In addition, research has indicated that ragweed pollen is likely to increase over much of Europe due to climate change with corresponding increases in ragweed allergy [4,5]. Across Europe, the population sensitised to ragweed is likely to more than double, from 33 to 77 million, by 2041–2060. This increase will occur where ragweed is a current health problem (e.g., Balkans and Hungary), but will also spread to countries were the health issues are less prevalent (e.g., France, Germany, Poland) [4]. In addition to an increase in ragweed sensitization, a longer pollen season is anticipated [4].

One method of addressing this threat is to improve our understanding of predisposing factors for allergic sensitisation to ragweed and disease. Such a study should specifically include potentially modifiable factors, such as environmental factors. Previous allergy research has identified a complex array of clinical and environmental variables as modulating factors in sensitization, which is an immune response characterized by the production of antibodies to a particular allergen, such as immunoglobulin E (IgE). These factors have been demonstrated to affect the development of allergic disease. These include genetic predisposing factors, including parental and familial allergy, and exposure to allergens (e.g., pollens, house dust mite, and pet dander), microbial compounds, and air pollutants. Some early life factors, such as smoking during pregnancy, and socioeconomic factors, such as affluence and level of education, have also been associated with the development of allergy [6]. Any assessment of risk factors for allergy sensitisation and disease must therefore integrate environmental and clinical risk factors and distinguish between the end points of sensitisation and discrete allergic diseases.

To the best of our knowledge, this is the first paper to explore the epidemiology of ragweed sensitisation in children with a particular focus on how a wide range of potentially modifiable environmental factors influence pediatric sensitisation to ragweed and the implications for allergic disease (wheeze, asthma, eczema, and rhinoconjunctivitis). Previous studies focused on the association between symptoms and ragweed pollen [7] and clinical risk factors in relation to sensitisation and disease [8]. In this study, we collected a wide range of information on indoor and outdoor environments as well as socio-economic data. This permitted us to (1) develop a risk factor profile for ragweed sensitization, (2) assess the implications for the childhood development of specific allergic diseases, and (3) highlight environmental modifications that may help decrease the spread of ragweed sensitisation and associated allergic disease across Europe.

To fully explore the role of environmental factors, this study focused on children. They are an important group because early sensitisation to allergens is a key risk factor for allergic disease [9,10] and early identification of sensitisation is important for the diagnosis and management of atopic (allergic) disease [11]. The scientific and medical communities have also expressed interest in the identification of specific IgE for early intervention to reduce the burden of subsequent allergic disease. Finally, children are an important research focus because they have less complex epidemiological histories than adults. This study of over 4000 children was conducted in Croatia, where some of the highest ragweed pollen concentrations in Europe have been recorded and where large regional variability in exposure exists [12].

## 2. Materials and Methods

Children (2–13 years) were recruited through primary schools and kindergartens (hereafter referred to as schools) in different regions of Croatia representing a range of ragweed exposure: Mediterranean (Dalmatia), Zagreb and surroundings (Central Croatia), and Slavonia (East Croatia). Schools within 15 km of the nearest ragweed pollen station were invited to participate in the study. Within each consenting school, parents and guardians were invited to a presentation of the study and were provided with written material about the study. Those who agreed to their children participating in the study completed a consent form. Children with chronic immunosuppressant or antihistamine therapy were excluded as these interfere with skin prick tests (SPT). In total, 4015 children (51% boys and 49% girls) were recruited between April 2012 and June 2014.

Children underwent SPTs with commercially available standard allergen extract (Alyostal ST-IR, Stallegenes S.A., France). In addition to ragweed, the children were tested with a standard panel of aeroallergens: pollens (birch, hazel, pine, olive, and a five-species grasses mix), dog dander, cat dander, house dust mite (*Dermatophagoides pteronyssinus*; HDM), *Parietaria*, and *Cladosporium*. Wheal size was recorded according to the International Study of Asthma and Allergies in Childhood (ISAAC) standardized protocol [13]. To provide objective measures of disease in children, parents were asked to complete a questionnaire based on ISAAC Phase II that contained questions regarding disease symptoms.

Ragweed sensitisation was defined as a wheal size of 2 mm or greater. Although some studies use a 3 mm threshold, a lower threshold is more appropriate in young children [14]. There is strong cross-reactivity within the Asteroideae subfamily, which includes ragweed (genus *Artemisia*), as well as some evidence of cross-reactivity of ragweed with pollen from grasses and trees [15]. Therefore, we performed a secondary analysis by removing children sensitised to another allergen with which ragweed may cross-react from the ragweed sensitised group. Disease presence (wheeze, asthma, eczema, and rhinoconjunctivitis) was categorised using the responses from the ISAAC Phase II questionnaire. Descriptions of the two sensitisation variables and the four disease variables are presented in Table 1.

The parental questionnaire also collected information on the home environment (e.g., fuel used for heating), lifestyle (e.g., smoking during pregnancy), family and personal medical history (e.g., familial disease), and socio-economic information (e.g., family income). The study protocol and informed consent, based on the international Declaration of Helsinki, Declaration on Human Rights and local legislation, were approved by the Local Ethics Committee (Children`s Hospital Srebrnjak, Croatia) and the E.U. ATOPICA project Ethics Committee.

A range of outdoor home environmental variables were collected. Using Geographical Information Systems (GIS) (ArcGIS 10.2, Redlands, CA, USA), each child was assigned to their nearest of eight pollen observation sites (Croatian Environment Agency) and associated long-term (2007–2012) ragweed pollen averages. This period was chosen as it precedes data collection and hence represents the period when sensitization would have occurred. Several stations were in close proximity, had similar pollen levels, and few children assigned to them. Therefore children were grouped into pollen areas to ensure that each contained at least 10% of the sample. This categorisation produced four ragweed pollen areas: Slavonski Brod (high pollen), Osijek (medium-high), Zagreb (medium), and Mediterranean (low) as shown in Table 2. 

Further outdoor home environmental information was obtained using GIS. Each child was assigned long-term (2007–2012) climate (temperature, precipitation) and air quality data (SO_2_, PM10, NO_2_, O_3_) from their nearest meteorological (European Climate Assessment and Dataset; [16], TuTiempo; [17]) and air quality (Airbase V8; [18]) observation site. The home elevation was calculated using SRTM (NASA Shuttle Radar Topographic Mission) data from CGIAR-CSI [19]. Circular neighborhoods (0.5 and 5 km) were assigned around each home and within these, the area of 44 different land-cover types were extracted using Corine Land Cover data [20]. Previous studies showed surrounding land use to be associated with pollen concentrations [21]. Within each neighborhood, the length of different categories of road, rail, and water features were calculated using OpenStreetMap [22]. The distance to the nearest of these features was obtained. Each home location was intersected with the Land Cover data. Any locations that intersected “artificial-surfaces” land cover were coded urban; the remaining were classified rural. For 165 children, the residential location was unavailable, so all these data were based upon school location. A full list of explanatory variables appears in supplementary materials, Table S1.

Data were analysed in STATA-13 (StataCorp, College Station, TX, USA) using clustered logistic regression with robust errors (clustering on school). The statistical analysis was undertaken to examine (1) the factors that affect sensitisation to ragweed pollen (all-ragweed sensitisation and restricted-ragweed sensitisation) and (2) influences on disease development (wheeze, asthma, eczema, and rhinoconjunctivitis). As all our explanatory variables were categorical or converted to a categorical variable, missing data were assigned to a unique category. A core model was produced including gender, age, and pollen area, and in the case of disease, the allergen-specific SPTs for other allergens to which the child might be sensitised, as a priori, these are important predictors of sensitisation and allergic disease. All other explanatory variables were added in turn to this core model and any that were significant (*p* < 0.1) were examined in more detail in a multivariable analysis. For the multivariable analysis, insignificant variables (*p* > 0.3) were eliminated in successive steps. In the final model, only variables significant at *p* < 0.05 were retained.

## 3. Results

Table 3 indicates that the most important factor affecting ragweed sensitisation (i.e., highest odds ratio) was the amount of ragweed pollen in an area. However, similar rates of sensitisation were observed in areas of medium, medium/high, and high levels of pollen, suggesting that sensitisation has reached a threshold in these areas. Despite the many environmental factors included in the model, only living in a rural area was significant and had a protective effect. Apart from pollen area, non-modifiable factors dominate Table 3. Ragweed sensitisation was lower in girls and increased with age. Protective factors included: being a second-born or subsequent child and use of antibiotics in the first year of life. Being sensitised to another aeroallergen and having a parent with allergic disease increased the risk of ragweed sensitisation. When the outcome variable was restricted, ragweed sensitisation, the pattern of sensitisation risk by pollen area, and having an atopic parent all intensified, meaning the odds increased relative to the reference category. As expected, being sensitised to other allergens had less influence (i.e., sensitised to other allergens that are not ragweed cross-reactive). The odds ratios (OR) for other variables remained similar but the significance levels decreased; this was anticipated given the smaller number of restricted ragweed sensitisations (Table 2).

Multivariable models were developed for each allergic disease and the results are shown in Table 4. The most important observation is that sensitisation to ragweed doubled the odds of rhinoconjunctivitis, but no association was observed between ragweed sensitisation and asthma, wheeze, or eczema. Over and above the effect of sensitisation in the case of rhinoconjunctivitis, no additional influence of ragweed pollen area was found on allergic disease. 

In addition to our consideration of ragweed, other sensitisations are associated with disease. House dust mite is positively associated with all types of allergic disease with the exception of eczema. Birch and grass sensitisation are strongly associated with rhinoconjunctivitis, grass sensitisation is associated with wheeze, and birch sensitisation displays associations with eczema. Cat and dog dander are associated with wheeze. In terms of other environmental factors, living in a rural area is associated with a lower risk of eczema.

For each disease, a range of non-environmental factors are also associated with disease development. Asthma and wheeze are significantly less prevalent in girls. For wheeze and rhinoconjunctivitis, a lowering of risk occurs with age. Rhinoconjunctivitis is lower after age four. Familial allergic disease is associated with an increased risk of wheeze, rhinoconjunctivitis, and eczema, whereas having an asthmatic parent is strongly associated with asthma. For all allergic diseases, having a respiratory infection in early childhood is a significant risk factor. Additionally, for asthma, having been treated for worms is a risk factor along with living in a home with more than two bedrooms. A higher family income is protective. For wheeze, sleeping on a feather pillow is protective alongside higher paternal education and living in a household with more than two adults. Maternal smoking in pregnancy is a risk factor. For rhinoconjunctivitis, not being the firstborn is protective.

## 4. Discussion

Europe is currently experiencing a ragweed invasion event and, in combination with climate change, sensitisation to ragweed is likely to double by 2041–2060. One method of addressing this threat is to improve our understanding of predisposing factors for allergic sensitisation to ragweed and disease, focusing specifically upon modifiable factors, such as indoor and outdoor environment and behavior. This is one of the first studies to explore the factors associated with ragweed sensitisation in children and its links to allergic disease. A unique feature of this study is its focus upon children who, because of their age, have less complex epidemiological histories.

Our results indicate that the risk of ragweed sensitisation was strongly associated with ragweed pollen levels. In Slavonski Brod, an area with high ragweed pollen concentrations (13,039 grains·m^−3^·year^−1^), the risk of sensitisation was nearly 16 times greater than in the Mediterranean area (422 grains·m^–3^·year^−1^). However, similarly high levels of sensitisation were also found in Zagreb (5188 grains·m^–3^·year^−1^) and Osijek (8275 grains·m^–3^·year^−1^). This indicates that levels of ragweed sensitisation have stabilised at around 5000 grains·m^–3^·year^−1^. Hence, to have an observable effect, strategies to reduce sensitisation should focus on reducing levels of pollen to below this level. Clearly, future research is required to determine the shape of the dose-response function and the duration of exposure required for sensitisation. One previous study focused on a diverse set of studies across Europe, suggested a logarithmic relationship between sensitisation and ragweed pollen, indicating that the curve is steepest between 0 and 2000 grains·m^–3^·year^−1^ [4]. The association between ragweed sensitisation and rhinoconjunctivitis is important as it causes not only nasal symptoms but also fatigue, sore throat, and cough [23]. In children, rhinoconjunctivitis can affect cognitive function [24]. No associations were found between ragweed sensitisation and the prevalence of asthma, wheeze, or eczema in children, and living in a high, medium (med), or low ragweed region. For asthma and wheeze, this aligns with previous research [25] that highlighted that the development of asthma is less strongly related to outdoor allergens such as ragweed.

The study included a number of indoor environment variables, such as pet ownership and the presence of carpeted floors, but again, these were all insignificant in the multivariable model for ragweed sensitisation or in the development of rhinoconjunctivitis. Our sample was diverse socioeconomically, yet all these variables were insignificant. Even the behavioral factor maternal smoking, accepted as a cause of allergic sensitisation [26], was surprisingly insignificant in spite of high rates of maternal smoking (22%) in our sample.

Using GIS, we created a number of outdoor environmental variables (e.g., proximity to railway lines, surrounding land use) that were hypothesised to be associated with localised ragweed exposure. None were significant, indicating that these measures confer no additional risk for children. Our results may also indicate that 500 m or 5 km circular buffers around a child’s home is a poor proxy for the locations where ragweed exposure may occur. The results also suggest that although distribution of ragweed plants may be associated with roads, rail lines, derelict sites, etc., the spatial distribution of airborne ragweed pollen to which the child (1–1.5 m height) is being exposed may not be associated with these land use features. Future studies may consider individual measures of exposure such as through global positioning systems (GPS) or mobile phone tracking [27,28]. In accordance with previous non-ragweed studies [29], living in a rural environment was protective against ragweed sensitisation. Unfortunately, due to the ecological nature of this study, we could not identify the elements of the rural environment that were protective. Previous non-ragweed studies have suggested as explanations lower traffic-related air pollution (air pollution was included in our models but sample variability was low), enhanced biodiversity of microbial exposure in rural and particularly farm environments, or co-exposure to environmental adjuvants (microbial and anthropogenic) [30].

In addition to ragweed exposure, most other drivers of sensitisation were weighted toward genetic and family factors that are difficult to modify. The excess risk found in the male children supports adult research [31]. Having an atopic parent is a risk factor. As common reported in non-ragweed studies [32], we found early life factors in infancy that were important for ragweed sensitisation and being the first-born child conferred a higher risk. However, the underlying biological mechanisms for this are unclear [33]. We found a protective effect of taking a course of antibiotics in the first year of life, whereas previous research has tended to find no association [34].

This study benefitted from a large sample size and high level of recruitment, implying that the sample is representative of the source populations. However, excluding a degree of selection bias is not possible as the study was voluntary in nature. Studies such as ours cannot determine whether the statistical associations observed are causal relationships or explore the chronology of exposure and disease. Our methods for assessing sensitisation and allergic disease questionnaires were based upon ISAAC protocols and methodologies, enhancing reliability and comparability with other studies. Although recall bias may have been an issue, empirical studies indicate that this is often nondifferential between cases and controls [35].

Our results suggest that the only effective strategy for controlling sensitisation to ragweed and the development of associated rhinoconjunctivitis is through control of ragweed pollen, as no other associated factors are potentially modifiable. This is a non-trivial task, and the most important factor is to prevent the spread of ragweed seed. This mostly occurs through anthropogenic processes such as transport of seed in other seed products and due to the transport of contaminated soil. Control measures again these processes are crucial [36]. When ragweed seed spreads, the plant does well on land that is regularly disturbed, implying that land management is an important element of control [3]. Control of plants is complex due to long-lived seeds, an ability to regrow after cutting, and an ability to become herbicide resistant [37]. Should sensitisation and allergy disease rates increase then disease management becomes important [7].

## 5. Conclusions

To the best of our knowledge, this is the first study to comprehensively research the epidemiology of ragweed sensitisation in children and the consequent implications for allergic disease. We focused on factors that are potentially modifiable. Results highlighted a saturation of ragweed sensitisation in children at pollen levels over 5000 grains·m^–3·^year^−1^ but shows few other potentially modifiable factors. The one exception was the protective effect of living in a rural area, but the reasons behind this association are unknown. Ragweed sensitisation is a risk factor for the development of rhinoconjunctivitis but no other allergic disease in children. Our results indicate that strategies to lower the risk of sensitisation and disease associated with ragweed should focus upon policies designed to reduce the spread and eradicate the ragweed plant.

## Figures and Tables

**Table 1:** Dependent variables of interest used in the study.

Ragweed Sensitised	Description	Prevalence (%)
Ragweed sensitisation	Wheal size from ragweed skin prick test (SPT) of 2 mm or greater	10.5
Restricted ragweed sensitisation	Wheal size from ragweed SPT of 2 mm or greater but not sensitised (SPT ≥ 2 mm) to birch, hazel, pine, olive, grass, and *Parietaria* pollen	4.6
Disease marker		
Wheeze	Positive response to the question “Has your child had wheezing or whistling in the chest in the last 12 months?”	17.4
Asthma	Positive response to “In the last 12 months, has your child had asthma?”	3.5
Eczema	Positive response to “Has your child had this itchy rash at any time in the last 12 months? AND positive response to the flexural eczema question “Has this itchy rash at any time affected any of the following areas: the folds of the elbows, behind the knees, in front of the ankles, under the buttocks, or around the neck, ears or eyes?”	18.3
Rhinoconjunctivitis	Positive response to “In the last 12 months, has your child had a problem with sneezing or a runny or blocked nose when he/she did not have a cold or the flu?” AND “In the last 12 months, has this nose problem been accompanied by itchy-watery eyes?”	38.9

**Table 2:** Ragweed pollen levels and children sensitised.

Categories of Ragweed Pollen Level	Average Total Annual Ragweed Pollen Grains m^3^	Pollen Area Name	Children Recruited		Ragweed Sensitised	Restricted Ragweed Sensitised
			Number	%	%	%
High	13,079	Slavonski Brod	540	13.45	15.19	6.04
Medium-high	8275	Osijek	582	14.50	17.53	7.41
Medium	5189	Zagreb	1324	32.98	15.79	9.97
Low	422	Mediterranean	1568	39.06	1.98	0.64
		Total	4014			

**Table 3:** Multivariable models for ragweed sensitised children.

Risk Factors		Ragweed Sensitised	Restricted RagweedSensitised
		Odds Ratio [CI ^+^]	Odds Ratio [CI ^+^]
Sex	Male	Ref	Ref
	Female	0.63 [0.52–0.77] ***	0.72 [0.54–0.96] *
Age (years) ^‡^	≤4	Ref	Ref
	5	1.24 [0.69–2.24]	1.54 [0.78–3.07]
	6	0.98 [0.54–1.76]	1.21 [0.67–2.19]
	7	1.33 [0.80–2.21]	1.38 [0.77–2.47]
	8	1.15 [0.80–2.21]	1.25 [0.69–2.27]
	9	1.73 [1.09–2.78] *	1.86 [1.09–3.19] *
	10	1.95 [1.04–3.66] *	1.47 [1.57–13.98]
Pollen area	Mediterranean (low)	Ref	Ref
	Zagreb (medium)	12.09 [7.31–20.00] ***	14.25 [6.08–33.38] ***
	Osijek (medium high)	16.81 [10.01–28.22] ***	25.01 [10.76–58.14] ***
	Slavonski Brod (high)	16.10 [9.62–26.95] ***	20.94 [8.34–52.60] ***
Sensitisation to other allergens	No	Ref	Ref
	Yes	11.05 [8.72–13.98] ***	2.56 [1.93–3.38] ***
Atopic parent ^‡^	No	Ref	Ref
	Yes	1.32 [1.02–1.69] *	1.48 [1.09–2.03] *
Child order ^‡^	First born	Ref	Ref
	Second or more	0.77 [0.60–0.98] *	0.70 [0.50–0.98] *
Use of antibiotics in first year of life ^‡^	No	Ref	Ref
	Yes	0.74 [0.58–0.93] *	0.73 [0.53–0.99] **
Urban/rural	Urban	Ref	Ref
	Rural	0.73 [0.55–0.98] *	0.75 [0.44–1.29]
Observations (*n*)		4009	3772

*** *p* < 0.001, ** *p* < 0.01, * *p* < 0.05. ^+^ 95% Confidence Intervals. ^‡^ a missing variable category was included for these variables. Ref = Reference Category.

**Table 4:** Factors associated with the development of allergic disease in children.

Risk Factorsheading		Asthma	Wheeze	Rhinoconjunctivitis	Eczema
		Odds Ratio [CI ^+^]	Odds Ratio [CI ^+^]	Odds Ratio [CI ^+^]	Odds Ratio [CI ^+^]
Sex	Male	Ref	Ref	Ref	Ref
	Female	0.46 [0.30–0.72] **	0.65 [0.54–0.79] ***	0.85 [0.70–1.03]	1.18 [1.00–1.38]
Age	4	Ref	Ref	Ref	Ref
	5	1.00 [0.43–2.34]	0.80 [0.64–1.02]	0.68 [0.52–0.90] **	0.92 [0.68–1.24]
	6	1.09 [0.57–2.08]	0.47 [0.36–0.62] ***	0.52 [0.36–0.74] ***	0.73 [0.52–1.02]
	7	1.47 [0.78–2.77]	0.42 [0.31–0.57] ***	0.52 [0.36–0.73] ***	0.71 [0.50–1.01]
	8	0.80 [0.38–1.55]	0.33 [0.24–0.44] ***	0.57 [0.42–0.78] ***	0.72 [0.52–0.99] *
	9	1.29 [0.60–2.77]	0.32 [0.23–0.44] ***	0.63 [0.47–0.83] **	0.79 [0.56–1.11]
	10	1.67 [0.77–3.63]	0.27 [0.17–0.44] ***	0.62 [0.39–0.99] *	0.86 [0.63–1.17]
Pollen Area	Mediterranean (low)	Ref	Ref	Ref	Ref
	Zagreb (medium)	1.44 [0.91–2.30]	0.79 [0.60–1.03]	1.13 [0.86–1.48]	0.84 [0.65–1.09]
	Osijek (medium high)	1.93 [1.00–3.70]	1.14 [0.85–1.53]	0.63 [0.44–0.92] *	0.71 [0.51 *–0.99] *
	Slavonski Brod (high)	0.96 [0.53–1.74]	1.31 [0.99–1.73]	1.34 [0.97–1.86]	0.88 [0.62–1.25]
Sensitisation to other allergens	Ragweed	1.61 [0.86–3.03]	1.18 [0.82–1.71]	2.17 [1.59–2.96] ***	1.39 [0.94–2.04]
	Dustmite	2.60 [1.60–4.24] ***	1.69 [1.29–2.22] ***	1.91 [1.52–2.40] ***	1.28 [1.00–1.64]
	Dog	1.52 [0.71–3.22]	1.61 [1.09–2.37] *	1.46 [1.00–2.15]	1.07 [0.72–1.60]
	Cat	1.97 [0.87–4.46]	1.65 [1.13–2.40] **	1.14 [0.77–1.70]	0.88 [0.58–1.33]
	Birch	0.81 [0.42–1.59]	1.08 [0.72–1.64]	2.31 [1.51–3.53] ***	2.08 [1.36–3.19] **
	Grass	1.64 [0.97–2.76]	1.48 [1.06–2.06] *	2.44 [1.86–3.21] ***	1.00 [0.72–1.38]
	Parietaria	0.33 [0.30–3.68]	1.16 [0.61–2.21]	0.93 [0.34–2.56]	1.86 [0.97–3.57]
Asthmatic parent ^‡^	No	Ref			
	Yes	3.34 [2.26–4.92] ***			
Has atopic family member ^‡^	No		Ref	Ref	Ref
	Yes		1.71 [1.37–2.12] ***	1.86 [1.51–2.29] ***	1.88 [1.54–2.29] **
Child has been treated by doctor for worms ^‡^	No	Ref			
	Yes	2.26 [1.09–4.69]*			
Respiratory infection in childhood ^‡^	No	Ref	Ref	Ref	Ref
	Yes	1.67 [1.13–2.47] *	1.83 [1.53–2.19] ***	1.54 [1.31–1.81] ***	1.30 [1.13–1.49] ***
Does child sleep on feather pillow ^‡^	No		Ref		
	Yes		0.80 [0.65–0.97] *		
Monthly household income ^‡^	<U.S. $660	Ref			
	>U.S. $660	0.52 [0.31–0.88] *			
Number of bedrooms in home ^‡^	1–2	Ref			
	>2	1.72 [1.12–2.64] *			
Maternal smoking in pregnancy ^‡^	No		Ref		
	Yes		1.40 [1.12–1.74] **		
Father’s education beyond high school diploma ^‡^	No		Ref		
	Yes		0.73 [0.57–0.93] *		
Number of adults in household ^‡^	1–2		Ref		
	>2		0.76 [0.57–1.00] *		
Child order ^‡^	Firstborn			Ref	
	Second child or more			0.80 [0.68–0.93] **	
Urban/rural	Urban				Ref
	Rural				0.73 [0.55–0.98] *
Observations (*n*)		3607	3620	3562	3597

*** *p* < 0.001, ** *p* < 0.01, * *p* < 0.05. ^+^ 95% Confidence Intervals. ^‡^ a missing variable category was included for these variables. Ref = Reference Category.

## Supplementary Materials

The following are available online at http://www.mdpi.com/1660-4601/15/7/1339/s1. Table S1. A full list of explanatory variables.
