# Supplementary material for: Modifiable Risk Factors for Common Ragweed (Ambrosia artemisiifolia) Allergy and Disease in Children: A Case-Control Study
Source: Int J Environ Res Public Health. 2018 Jun 26;15(7):1339. doi: 10.3390/ijerph15071339 (PMC6069153; doi:10.3390/ijerph15071339)
Supplement: Supplementary file 1 [file ijerph-15-01339-s001.zip › ijerph-314139-SI.pdf]

Table S1: Explanatory variables used in analysis

Variable Name	Variable label
**ID_SPT**	Child Identity
**Area**	Original pollen areas (4)
**pollen_Area**	Mean total pollen by Area
**pollen_nearest_code**	Nearest pollen station to child home location
**pollen_totalmean**	mean pollen total mean by station
**DateOfBirth**	Date of birth: Day (dd) of birth
**Age**	Age reported
**Gender**	Gender
**School_ID**	School Identification Number
**control_pos**	SPT wheal size result: (+) Control/Histamine (mm)
**control_neg**	SPT wheal size result: (-) Control (mm)
**Trees**	SPT wheal size result: Trees mixtures (mm)
**Birch**	SPT wheal size result: Birch (mm)
**Hazel**	SPT wheal size result: Hazel (mm)
**Grass**	SPT wheal size result: Grasses mix (mm)
**Amb**	SPT wheal size result: Ambrosia (mm)
**Dog**	SPT wheal size result: Dog hair (mm)
**Cat**	SPT wheal size result: Cat fur (mm)
**Dustmite**	SPT wheal size result: D Pteronyssimus (mm)
**Clado**	SPT wheal size result: Cladosporium (mm)
**Pine**	SPT wheal size result: Pine (mm)
**Olive**	SPT wheal size result: Olive (mm)
**Parietaria**	SPT wheal size result: Parietaria (mm)
**resp_infect_y1**	How often has your child had respiratory infections during the first year of life?
**resp_infect_y2**	How often has your child had respiratory infections during the second and third year of life?
**fever_y1**	How often, during the first year of life, has your child had fever (higher than 38.5 °C)?
**fever1**	Child had fever higher than 38.5 °C during 1 year of live
**med_temp**	When do you give your child medicine for lowering body temperature?
**anti_y1**	How often, during the first year of life, did your child take antibiotics for more than 3 days in a row?
**anti_y23**	How often, during the second and third year of life, did your child take antibiotics for more than 3 days in a row?
**anti1**	Child had taken antibiotic s for more than 3 days in a row during 1 year of life
**worms**	Has your child ever been treated by the doctor because of abdominal pain caused by worm infection?
**nursery**	Did your child ever go to a child care facility or nursery school?
**nursery_start**	If yes (Q39), from ____ years of age? (in months)
**nursery_end**	If yes (Q39), till ___ years of age? (in months)
**kinder**	Did your child ever go to a kindergarten?
**kinder_start**	If yes (Q40), from ____years of age? (in months)
**kinder_end**	If yes (Q40), till ___ years of age? (in months)
**nurs_kind_length**	Length of time (months) in nursery / kindergarten
**nurs_kind**	Percentage of life (or first seven years if older than 84 months) spent in either nursery or kindergarten
**multibirth**	Is your child a twin, or a triplet/quadruplet?
**multibirth_num**	If yes (Q41), a twin, triplet, quadruplet?
**sibling_count**	How many brothers and sisters does your child have?
**brothers**	How many brothers?
**sisters**	How many sisters?
**child_order**	Where does your child come in the family (firstborn, second….)?
**siblings**	number of siblings the child has (0, 1 or more)
**atopic_fam**	Did any of your close family members had/s/ll have any atopic diseases, like asthma, allergic rhinitis or atopic dermatitis?

**atopic_fam_adj**	Did any of your close family members had/s/ll have any atopic diseases, like asthma, allergic rhinitis or atopic dermatitis?
**atopic_mem**	If yes (Q44), which family members?
**asth_bro**	How many brothers do/did suffer from asthma?
**rhin_bro**	How many brothers do/did suffer from allergic rhinitis?
**ecz_bro**	How many brothers do/did suffer from atopic dermatitis?
**atopic_bro**	Has brother with asthma or allergic rhinitis or atopic dermatitis
**asth_sis**	How many sisters do/did suffer from asthma?
**rhin_sis**	How many sisters do/did suffer from allergic rhinitis?
**ecz_sis**	How many sisters do/did suffer from atopic dermatitis?
**atopic_sis**	Has sister with asthma or allergic rhinitis or atopic dermatitis
**asth_mum**	Has the child's mother ever had asthma?
**rhin_mum**	Has the child's mother ever had allergic rhinitis?
**ecz_mum**	Has the child's mother ever had atopic dermatitis (neurodermatitis, eczema)?
**atopic_mum**	Has mother with asthma or allergic rhinitis or atopic dermatitis
**asth_dad**	Has the child's father ever had asthma?
**rhin_dad**	Has the child's father ever had allergic rhinitis?
**ecz_dad**	Has the child's father ever had atopic dermatitis (neurodermatitis, eczema)?
**atopic_dad**	Has father with asthma or allergic rhinitis or atopic dermatitis
**atopic_parent**	Has atopic father or mother?
**atopic_parent2**	Has atopic father or mother?
**Asth_PARENT**	Any parent has/had asthma
**Rhin_PARENT**	Any parent has/had Rhinitis
**Ecz_PARENT**	Any parent has/had Eczema
**dog_home**	Do/did you keep a dog as a pet in your home?
**cat_home**	Do/did you keep a cat as a pet in your home?
**ham_home**	Do/did you keep a hamster as a pet in your home?
**bird_home**	Do/did you keep birds as a pet in your home?
**dog_contact**	Did your child have regular contact with dogs (e.g., at your friend's home)?
**cat_contact**	Did your child have regular contact with cats (e.g., at your friend's home)?
**ham_contact**	Did your child have regular contact with hamsters (e.g., at your friend's home)?
**bird_contact**	Did your child have regular contact with birds (e.g., at your friend's home)?
**pet**	Child has/had contact cat, dog or hamster or at home
**bird**	Child has/had contact or bird at home
**poultry**	Did your child have regular contact with poultry?
**pigs**	Did your child have regular contact with pigs?
**cows**	Did your child have regular contact with cows?
**horses**	Did your child have regular contact with horses?
**rabbits**	Did your child have regular contact with rabbits?
**other_animals**	Did your child have regular contact with any other animal?
**AnimalFarm**	Child has/had contact with a farm animal/ Farm (only no-yes)
**sm_preg**	Did your child's mother smoke during pregnancy with your child?
**cig_count_preg**	If yes (Q51), how many cigarettes a day?
**sm_home**	Does anybody, at present, smoke inside your child's home?
**sm_home_freq**	If yes (Q52): how often did they smoke / during which stage of your child's life?
**cig_home10**	Are less than 10 cigarettes in total smoked per day in the child's home?
**cig_home20**	Are10-20 cigarettes in total smoked per day in the child's home?
**cig_home20plus**	Are > 20 cigarettes in total smoked per day in the child's home?
**sm_preg2**	Mother smoking during pregnancy (recoded 1= yes + periodically)
**SMOKING**	Smoking during pregnancy or smoking at home
**breastfed**	Was your child breast fed?
**breastfed_time**	If yes (Q54), for how long?
**preg_anti**	How often, during the pregnancy, was the child's mother on the antibiotics for more than
**feather**	Does or did your child sleep on a feather pillow?
**feather_freq**	How often does your child sleep on a feather pillow / during which stage of life?
**mattage_y1**	How old was the mattress that was used by your chi during the first year of life?

**mattage_y27**	How old was the mattress that was used by your child from 2nd till 7th year of life?
**mattage_after7y**	How old was the mattress that was used by your chi a.er 7th year of life?
**mattage**	mattress older than 3 years at any stage of child life
**carpet**	Is/was there a fitted carpet in your child's bedroom?
**carpet_freq**	Frequency of fitted carpet in child's bedroom / stage of life?
**gas**	Do or did you use gas for heating in your child's home?
**oil**	Do or did you use oil for heating in your child's home?
**elec**	Do or did you use electricity for heating in your child's home?
**wood**	Do or did you use wood for heating in your child's home?
**coal**	Do or did you use coal/coke for heating in your child's home?
**cen_heat**	Do or did you use central heating in your child's home?
**gas1**	Gas heating (only no-yes)
**oil1**	Oil heating (only no-yes)
**elec1**	Electric heating (only no-yes)
**wood1**	Wood heating (only no-yes)
**cen_heating1**	Central heating (only no-yes)
**ed_mum**	Mother: What is the highest level of education you have completed?
**ed_dad**	Father: What is the highest level of education you have completed?
**employ_m**	Mother is (employment status):
**employ_d**	Father is (employment status):
**Placename_angl**	What town do you live in?
**urban_rural_adj**	Is the area you live in (rural/urban): (SEQ)
**population_adj**	If you live in an urban area, what is the population number?
**populationCat**	Population quintiles
**farm**	If you live in a rural area, do you live on a farm?
**income**	Including wages, salaries, self-employment, and any other source of income, was the total combined family income during the one month period?
**adults_home**	How many adults live in your household?
**kids_home**	How many children live in your household?
**bedrooms**	How many bedrooms are in your household (including children's room and guest rooms)?
**BEDROOMS**	number of rooms in household
**airq_nearest_code**	Air quality station nearest to child home location
**O3_nearest_code**	O3 station nearest to child home location
**Weather_nearest**	Weather station nearest to child home location
**Meantotal**	Mean of total ragweed pollen counts/m3 per year
**Meanmax**	Mean of maximum daily ragweed pollen counts/m3
**days5**	number of days>5 pollen counts/m3
**days10**	number of days>10 pollen counts/m3
**days15**	number of days>15 pollen counts/m3
**days20**	number of days>20 pollen counts/m3
**days25**	number of days>25 pollen counts/m3
**days30**	number of days>30 pollen counts/m3
**days50**	number of days>50 pollen counts/m3
**days100**	number of days>100 pollen counts/m3
**days200**	number of days>200 pollen counts/m3
**days500**	number of days>500 pollen counts/m3
**days1000**	number of days>1000 pollen counts/m3
**SO2_mean_d**	SO2_mean_d ug/m3 daily mean
**SO2_mean_h**	SO2_mean_h ug/m3 hourly mean
**PM10_mean_d**	PM10_mean_d ug/m3 daily mean
**PM10_mean_h**	PM10_mean_h ug/m3 hourly mean
**NO2_mean_d**	NO2_mean_d ug/m3 daily mean
**NO2_mean_h**	NO2_mean_h ug/m3 hourly mean
**O3_mean_d**	O3_mean_d ug/m3 daily mean
**O3_mean_h**	O3_mean_h ug/m3 hourly mean
**SO2_Area**	Mean hourly SO2 by Area

**PM10_Area**	Mean hourly PM10 by Area
**O3_Area**	Mean hourly O3 by Area
**NO2_Area**	Mean hourly NO2 by Area
**PT_DJF_19902010**	PT_DJF_1990-2010 Precipitation mm Dec, Jan, Feb
**PT_MAM_19902010**	PT_MAM_1990-2010 Precipitation mm Mar, Apr, May
**PT_JJA_19902010**	PT_JJA_1990-2010 Precipitation mm Jun, Jul, Aug
**PT_SON_19902010**	PT_SON_1990-2010 Precipitation mm Sep, Oct, Nov
**TG_DJF_19902012**	TG_DJF_1990-2010 Mean daily temperature C Dec, Jan, Feb,
**TG_MAM_19902012**	TG_MAM_1990-2010 Mean daily temperature C Mar, Apr, May
**TG_JJA_19902012**	TG_JJA_1990-2010 Mean daily temperature C Jun, Jul, Aug
**TG_SON_19902010**	TG_SON_1990-2010 Mean daily temperature C Sep, Oct, Nov
**Elevation**	Elevation m
**Continuousurbanfabric**	Continuous urban fabric m2
**Discontinuousurbanfabric**	Discontinuous urban fabric m2
**Industrialorc~**	Industrial or commercial units m2
**Roadandrailne~**	Road and rail networks and associated land m2
**Portareas**	Port areas m2
**Airports**	Airports m2
**Mineralextrac~**	Mineral extraction sites m2
**Dumpsites**	Dump sites m2
**Constructionsites**	Construction sites m2
**Greenurbanareas**	Green urban areas m2
**Sportandleisu~**	Sport and leisure facilities m2
**Nonirrigateda~**	Non-irrigated arable land m2
**Permanentlyir~**	Permanently irrigated land m2
**Ricefields**	Rice fields m2
**Vineyards**	Vineyards m2
**Fruittreesand~**	Fruit trees and berry plantations m2
**Olivegroves**	Olive groves m2
**Pastures**	Pastures m2
**Annualcropsas~**	Annual crops associated with permanent crops m2
**Complexcultiv~**	Complex cultivation patterns m2
**Landprincipal~**	Land principally occupied by agriculture, with significant areas of natural vegetation m2
**Agroforestrya~**	Agro-forestry areas m2
**Broadleavedf~s**	Broad-leaved forest m2
**Coniferousfor~**	Coniferous forest m2
**Mixedforest**	Mixed forest m2
**Naturalgrassl~**	Natural grasslands m2
**Moorsandheath~**	Moors and heathland m2
**Sclerophyllou~**	Sclerophyllous vegetation m2
**Transitionalw~**	Transitional woodland-shrub m2
**Beachesduness~**	Beaches, dunes, sands m2
**Barerocks**	Bare rocks m2
**Sparselyveget~**	Sparsely vegetated areas m2
**Burntareas**	Burnt areas m2
**Glaciersandpe~**	Glaciers and perpetual snow m2
**Inlandmarshes**	Inland marshes m2
**Peatbogs**	Peat bogs m2
**Saltmarshes**	Salt marshes m2
**Salines**	Salines m2
**Intertidalflat**	Intertidal flats m2
**Watercourses**	Water courses m2
**Waterbodies**	Water bodies m2
**Coastallagoons**	Coastal lagoons m2
**Estuaries**	Estuaries m2

**Seaandocean**	Sea and ocean m2
**abandoned_5km**	abandoned_5km m
**disused_5km**	disused_5km m
**abandoned_or_~**	Abandoned or disused 5 km buffer m
**rail_5km**	rail_5km m
**tram_5km**	tram_5km m
**living_street**	living_street m
**motorway**	motorway m
**path**	length of path in m
**pedestrian**	length of pedestrian in m
**platform**	length of platform in m
**primary**	length of primary roads m
**residential**	length of residential roads in m
**road**	length of road in m
**secondary**	length of secondary roads m
**service**	length of service road in m
**tertiary**	length of tertiary roads m
**trunk**	length of trunk roads m
**unclassified**	length of unclassified roads m
**distancetonea~**	distance to nearest motorway m
**distan~maryroa**	distance to nearest primary road m
**distan~daryroa**	distance to nearest secondary road m
**distan~iaryroa**	distance to nearest tertiary road m
**river**	length of rivers m
**stream**	length of streams m
**all_water_bod~**	All water bodies
**water**	Waterbodies + Watercourses
**waterCat**	water thirds if wb >0
**ALLROADdistance**	distance to primary, secondary or tertiary road
**rural_urban_code**	Rural or urban location of child home (GIS)
**VegArtificial**	Sportandleisurefacilities + Greenurbanareas
**UrbanRoad**	distance to roads in urban and rural areas
**UrbanRoad5**	distance to primary, secondary or tertiary roads in urban and rural areas
**UrbanRoad4**	distance to primary, secondary or tertiary roads in urban and rural areas
